# Utilization of discarded face masks in combination with recycled concrete aggregate and silica fume for sustainable civil construction projects

**DOI:** 10.1038/s41598-023-50946-z

**Published:** 2024-01-03

**Authors:** Fahad Amin, Muhammad Faisal Javed, Imtiaz Ahmad, Osama Asad, Nangyal Khan, Abdul Basit Khan, Shahid Ali, Sherzod Abdullaev, Fuad A. Awwad, Emad A. A. Ismail

**Affiliations:** 1 Department of Civil Engineering, COMSATS University Islamabad, Abbottabad Campus, Islamabad, Pakistan; 2 Department of Electronics Engineering, Peking University, Beijing, 100871 China; 3 Engineering School, Central Asian University, Central Asian University, Tashkent, Uzbekistan; 4 Scientific and Innovation Department, ashkent State Pedagogical University named after Nizami, Tashkent, Uzbekistan; 5 Department of Quantitative Analysis, College of Business Administration, King Saud University, P.O. Box 71115, 11587 Riyadh, Saudi Arabia

**Keywords:** Computational platforms and environments, Data integration

## Abstract

The coronavirus (COVID-19) pandemic has not only had a severe impact on global health but also poses a threat to the environment. This research aims to explore an innovative approach to address the issue of increased waste generated by the pandemic. Specifically, the study investigates the utilization of discarded face masks in combination with recycled concrete aggregate (RCA) and Silica Fume (SFM) in civil construction projects. The disposable face masks were processed by removing the ear loops and nose strips, and then cutting them into small fibers measuring 20 mm in length, 5 mm in width, and 0.46 mm in thickness, resulting in an aspect ratio of 24. Various proportions of SFM and RCA were incorporated into the concrete mix, with a focus on evaluating the compressive strength, split tensile strength, and durability of the resulting material. The findings indicate that the addition of SFM led to improvements in both compressive and split tensile strength, while no significant impact on durability was observed.

## Introduction

The Coronavirus (COVID-19) pandemic has caused an enormous increase in the use of personal protective equipment (PPE) which includes facemasks and plastic gloves^1^. It was mandatory to wear a facemask in public in many countries. Facemask usage by the general population contributed to reducing the spreading of the disease but on another hand, it is causing an increase in pollution. The presence of these PPE i.e. facemasks in large amounts has been confirmed by the divers in the bed of the rivers and oceans^2–4^. An estimated amount of daily facemask use in Africa is 7 hundred million, while in Asia the daily facemask usage number shows 2.2 billion^5–7^. In June 2020 the monthly number of facemasks discharged to the environment was estimated to be 129 billion^8^. A total of 6.88 billion facemasks weighing approximately 206,407 tons are globally generated and then dumped into landfills daily^5^. Due to an increase in littering, these used facemasks can be found practically anywhere, including on streets, in parking lots, and in neighborhood parks. The light weight of the facemasks makes it possible for wind and rain to successfully transport them from the landfills or garbage bins into the city streets, rivers, and seas, where the plastic-based masks can break down into microplastics. The primary plastic material used in single-use surgical masks, polypropylene, is a thermoplastic polymer that takes more than 25 years to degrade in landfills^9^. However, when single-use surgical masks are flown into the water streams, they break down into microplastics that could reach our food supply^10–12^. As a result, the pandemic’s impacts are not only having a significant negative impact on our economy but will also last long after the pandemic has passed. The non-biodegradable nature of the material of the facemask causes it to take more than 450 years to break down into the environment^13^. Since plastic fibers have several sustainability advantages over steel reinforcement, they have long drawn interest from the academic community and the building sector^14^. Due to its mechanical characteristics, such as tensile strength and Young's modulus, as well as its ease of manufacturing and high alkaline resistance, polypropylene fibers (PP) are widely employed across the concrete industry^15,16^. Previously Islam and Gupta (2016) analyzed polypropylene fiber-reinforced concrete in an experiment by adding these fibers between 0 and 0.3% (by volume of concrete) of polypropylene in the concrete mix. It was observed that the addition of polypropylene fibers by volume is causing a bit of drop in compressive strength throughout the testing period. Although the compressive strength decreased, the splitting tensile strength significantly increased when polypropylene fibers were added, increasing by 39% with the addition of 0.1% fibers by volume^17^.

One of the key ingredients in concrete is aggregates, both coarse and fine which play a significant influence in determining the overall technical qualities of concrete. The massive depletion of non-renewable natural resources had negative economic and environmental effects as a result of the heavy use of natural resources brought on by the constant high demand for new infrastructure and buildings^18^. The demolition and construction debris is made up of a diverse range of resources, including recovered aggregate, reclaimed asphalt, crushed brick, crushed glass, and crushed rock^19–21^. The construction and demolition waste constitutes about 45% of globally generated waste^13^. A literature review made by Tam found out By comparing data from 24 significantly developed nations across five continents, the authors were able to demonstrate how eager these nations are to encourage and promote the use of various recycled aggregates^22^. These nations have done this by passing new laws and raising awareness in the media about the need to reduce landfills and the extraction and consumption of natural mineral resources^22^. Recycling concrete debris is becoming increasingly important and is one of the most promising solutions to both shortages of natural resources and recycling of solid waste product materials because concrete is the second most abundant material on Earth, the first most used in construction and infrastructure, and the primary customer of non-renewable materials^23^. Crushing these concrete chunks into small pieces results in Recycled concrete aggregate (RCA) which can be used for different application like Buildings, pavements and for other constructions. RCA of 20 mm nominal size is used generally in pavement applications^24–26^.

Ordinary Portland cement is widely used in the construction sector and is one of the main sources of CO_2_ emissions, which harms the environment^11^. The emission of CO_2_ from the usage of ordinary Portland cement (OPC) caused pollution to the environment and affects the health of all living beings^18^. The construction industry will soon take action on the problem of decreasing its carbon output, which now ranges from 25 to 40% of emissions and of which just the production of cement accounts for 7–8%^27^. By using green building materials during construction and renewable energy during building operations, attempts are being made to have the whole construction sector reach net-zero carbon emissions by the year 2050^27^. To replace ordinary Portland cement in concrete, alternative materials have been introduced to lower the consumption of OPC in concrete.

Silica Fume is a by-product produced from the manufacture of silicon metal or Ferrosilicon Alloys, which is widely available in the world. Additionally, the use of Silica Fume is more environmentally friendly and save cost compared to OPC^28^. Initially, the silica fume remains inert when added to concrete^29–31^. After the start of the reaction between water and Portland cement, hydration starts, and two chemical compounds are formed as a result of this primary reaction: calcium silicate hydrate (CSH) the strength producing crystallization, and a by-product calcium hydroxide (CH), this is also known as free lime whose basic purpose is only as filler for lining the available pores within concrete. Calcium hydroxide reacts with the silica fume resulting in a Pozzolanic reaction leading to the production of additional CSH in many of the voids around hydrated cement particles. This results in increased compressive strength, flexural strength ^28^, and bonding strength ^32^ along with the formation of a denser matrix preferably in those areas which would have remained as small voids subject to possible ingress of deleterious materials. Silica fume increasing percentage reduces the porosity which increases the compressive strength^33^.

The previous studies on these wastes (SFM, RCA) show a decrease in the mechanical properties of concrete. However, this research carried out a series of experiments to reduce environmental waste and to evaluate different percentages of SFM and RCA for improving the mechanical properties of concrete by introducing silica fume as an additional binder into different mixes. Based on literature review and previous studies, none of the existing studies assessed these wastes in concrete with the use of Silica fume in different mixes according to the author’s knowledge. To fill this research gap silica fume is introduced to increase the compressive strength as well as the density of concrete. Several tests have been carried out to check the compressive strength, tensile strength of the concrete under various mix designs. Moreover, concrete having optimum percentages of SFM and RCA, durability of the concrete have been tested as it is also been revealed by the previous studies that to improve durability of concrete use fibers^34,35^.

## Material and methods

The basic material that is taken under consideration to investigate is concrete consisting of SFM fibers (Polypropylene). In this research new Single use facemasks were used due to COVID-19 precautions. In this section firstly different properties of the materials (SFM, RCA, Silica Fume, and Concrete) are presented, in the second part of the section, a description and composition for the concrete experimental research are given. To verify properties, 3 samples of each result are tested during research and all the numeral findings shows the average result of those 3 samples.

### Shredded face mask (SFM)

To carry out this research, new disposable surgical face masks were employed, featuring polypropylene fibers arranged in a random orientation and subsequently compressed and were fused into a pattern of small, closely spaced welds. Before using a single-use face mask in concrete, preparation of the masks is a must. Initially, the ear straps and all other metal parts were removed manually. Then the masks were cut down into smaller sizes manually. The fibers can be used in two forms i.e., short fibers and long fibers. In this research short fibers were used due to their easiness in handling and dispersion having length, width, and thickness of 20 mm, 5 mm, and 0.46 mm respectively. The aspect ratio is the length-to-diameter ratio of fiber, for short fibers, the aspect ratio ranges from 20 to 60, while for long fibers it is more than 200. According to *Naaman (2003)*, the aspect ratio of a fiber with a non-circular cross-section can be calculated as:

$${\text{Aspect}}\,{\text{ratio}} = \frac{l}{{d{\text{FIER}} }} = \frac{l}{{4\frac{A}{{\Psi }}}}$$

where l is the length, d_FIER_ is the equivalent diameter, A is the cross-sectional area, and Ψ is the perimeter of the fiber cross-section. The aspect ratio was approximately 24 ^36^.

**Figure 1:**
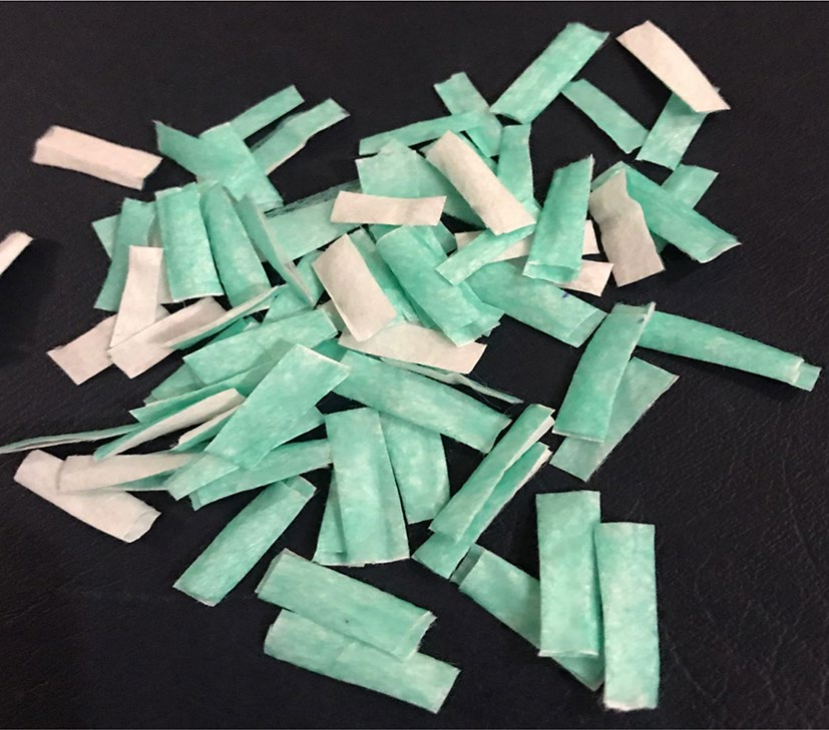
SFM fibers.

**Figure 2:**
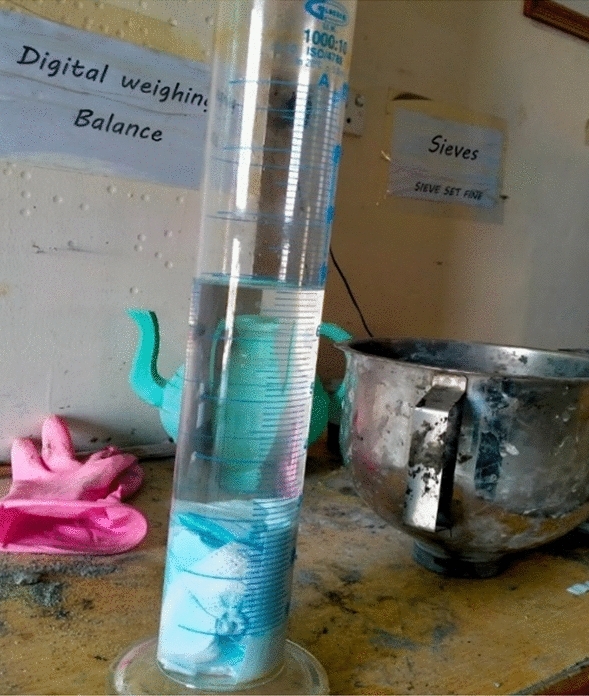
Cylinder with facemask depth.

The Density of a single-use Facemask was found by taking a cylinder filled with water up to 600 ml and then a 30gm (0.030 kg) of facemask sample was immersed in the cylinder for 24 h, after 24 h all the air voids were removed from the cylinder and the water level reached to 680 ml. The volume change was recorded to be 80 ml (0.00008 m^3^), from the formula of mass per unit volume, the Density was achieved as shown in Table 1. The procedure for calculating density is explained in Figs. 1 and 2.

**Table 1:** Density of single-use facemask.

Ingredients	Mass (gm)	Volume of cylinder (ml)
Water	–	600
Water and facemask	–	680
Change in volume	–	80
Facemask	30	80
Density (kg/m^3^)		370

### Recycled concrete aggregates (RCA)

This research work was carried out by using materials that include water, cement, and aggregates. RCA was brought from the demolished road shoulders for sewage work in Abbottabad and was reduced to a nominal size of 19 mm by crushing the old concrete junks manually. The strength of the old concrete junks was in the range of 20–25 MPa. It is known that normal concrete is composed of 75% of coarse aggregates for which some mechanical and physical properties are required to be determined. These properties of natural and recycled concrete aggregates were determined according to the relevant ASTM standards shown in Table 2

**Table 2:** Physical and mechanical properties of aggregates.

Properties of aggregates	Aggregate size	Standards	Natural aggregates	Recycled concrete aggregates
Specific gravity (gm/cm^3^)	Fine	ASTM C128-15	2.66	2.42
	Coarse	ASTM C127-15	2.65	
Water absorption	Fine	ASTM C128-15	3%	7.4%
	Coarse	ASTM C127-15	0.81%	
Moisture content	Fine	ASTM C566-13	3.28%	6.7%
	Coarse	ASTM C566-13	1.99%	
Impact test	Coarse	ASTM-D256	18.51%	16.17%
Fineness modulus	Fine	ASTM-C117-05	3.12	–

### Silica fume

Taking into consideration the effects of silica fume in enhancing the properties of concrete, along with protecting the environment by using this by-product in the construction industry rather than its disposal to the environment, an addition of 10% by weight of ordinary Portland cement in the mix was used which was taken from factory “Construction Chemicals & Services” located in Rawalpindi, Pakistan. A large proportion of the silica fume particles (95%) are finer than 1 µm. This nature and particle size of silica fume allows the concrete mix to become denser by filling the voids that are caused by using the SFM fibers. The reduction in porosity is more when silica fume was added up to a 10% level. Above 10%, the reduction in porosity is negligible.

### Concrete

Generally, concrete is composed of 75% coarse aggregates whose properties are defined in Table 3. The rest 25% includes water, cement, and fine aggregates. Water from COMSATS University Abbottabad was used for concrete production and curing. Ordinary Portland cement was used whose physical and chemical properties are given in Table 4 and the specific gravity was 3.15. Natural aggregates were brought from Thandiani near Abbottabad. The properties of aggregate are presented in Table 3.

**Table 3:** Physical and mechanical properties of aggregates.

Properties of aggregates	Aggregate size	Standards	Natural aggregates	Recycled concrete aggregates
Specific gravity (gm/cm^3^)	Fine	ASTM C128-15	2.66	2.42
	Coarse	ASTM C127-15	2.65	
Water absorption	Fine	ASTM C128-15	3%	7.4%
	Coarse	ASTM C127-15	0.81%	
Moisture content	Fine	ASTM C566-13	3.28%	6.7%
	Coarse	ASTM C566-13	1.99%	
Impact test	Coarse	ASTM-D256	18.51%	16.17%
Fineness modulus	Fine	ASTM-C117-05	3.12	–

**Table 4:** Chemical compositions of the cement (%), and mechanical and physical properties.

1	SiO_2_	20–20.5	
2	Al_2_O_3_	5.5–6	
3	Fe_2_O_3_	4.5–6	
4	CaO	63–64	
5	MgO	3–5	
6	C_3_A	7.5–8	
7	SO_3_	2.5–3	
8	Cl	0.1–1	
9	Compressive strength (MPa)	3 day	120–170
		7 day	200–280
		28 day	325-
10	Initial setting (min.)	45	
11	Final setting (min.)	360	

With a target compressive strength of 25 MPa, recommended slump value of 20–30 mm, and maximum aggregate size of 19 mm having a W/C ratio of 0.62, the control concrete mix design based on the ACI method was formed. Samples were formed in which the natural aggregates were replaced by 15%, 25%, and 50% RCAs. RCAs absorb more water than natural aggregates. Due to higher water absorption of the RCA, an excessive amount of water in the mixture was taken into account. Additionally, Silica fume was used 10% by weight of cement and SFMs as 1%, 2%, and 3% by volume of concrete. All the trail mixes and control sample mix design is given in Table 5.

**Table 5:** Mix design.

Mix No.	w/c	Water (kg/sample)	Cement (kg/sample)	Silica Fume (kg/sample)	Fine agg. (kg/sample)	Coarse Agg. (kg/sample)	Recycled concrete agg. (%)	Weight (kg/sample)	Vol. Facemask (%)	Weight (g/sample)
A1										
1	0.62	1.007	2.097		3.93	5.08	–	–	–	–
2	0.62	1.007	2.097	0.209	3.93	5.08	–	–	1	19.6
3	0.62	1.007	2.097	0.209	3.93	5.08	–	–	2	39.2
4	0.62	1.007	2.097	0.209	3.93	5.08	–	–	3	58.8
B1										
5	0.62	1.007	2.097	0.209	3.93	4.32	15	0.76	1	19.6
6	0.62	1.007	2.097	0.209	3.93	3.816	25	1.27	1	19.6
7	0.62	1.007	2.097	0.209	3.93	2.54	50	2.54	1	19.6

### Compressive strength test

**Figure 3:**
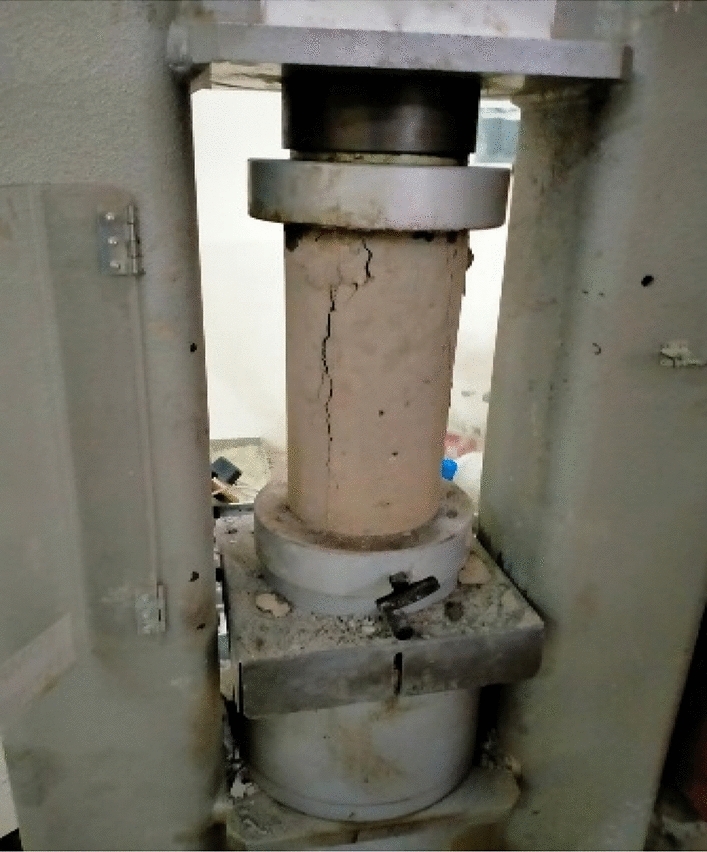
Compressive strength test.

The compressive strength tests were performed on cylindrical shape samples of dimensions (diameter and height) 150 mm and 300 mm according to ASTM-C39. The samples were tested on different curing Days i.e. 7, 14, and 28 days. The samples were subjected to loading until it breaks and the corresponding strengths were noted down as shown in Fig. 3.

### Splitting tensile strength

**Figure 4:**
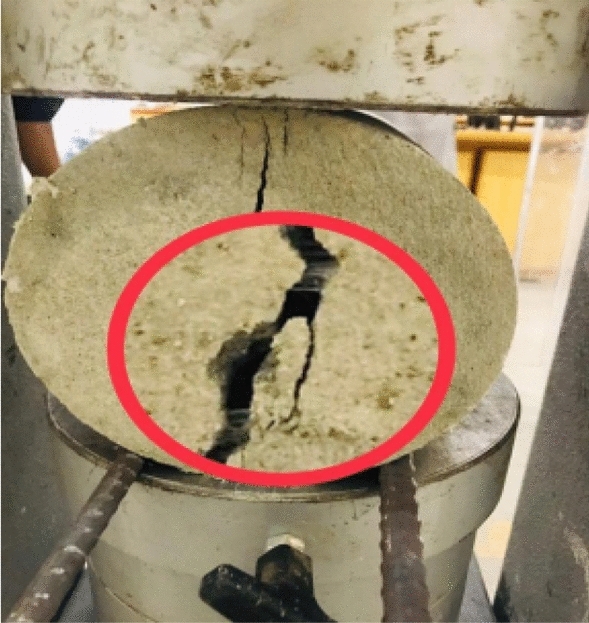
Bridging effect of fiber.

Acquiring the results from the compressive strength test, the specimens with the highest compressive strength were subjected to diametral loading to get the splitting tensile strength for 28 days according to ASTM C496. The specimen of cylindrical shapes having standard dimensions i.e. 150 mm in diameter and 300 mm in height, were subjected to loading along the diameter of the specimen until it’s splitting apart under a loading rate of 1.2–2.4 MPa/min as shown in Fig. 4.

### Durability

**Figure 5:**
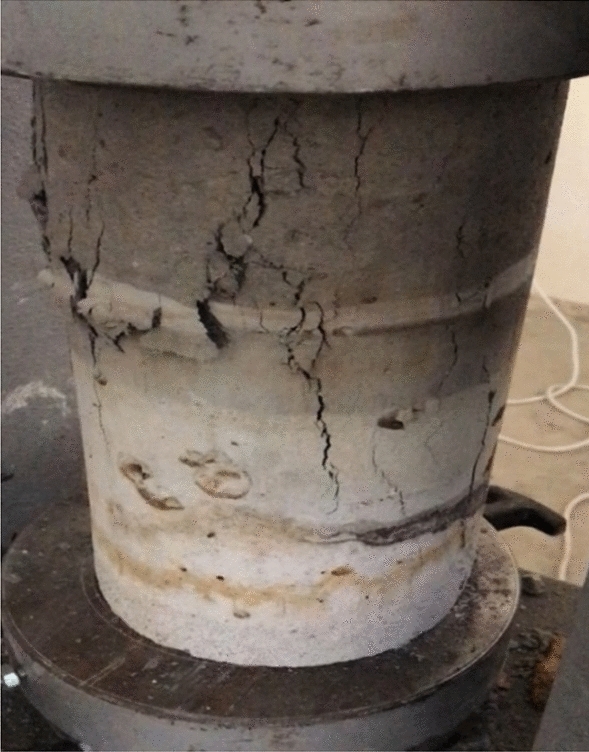
Durability test sample.

For durability of concrete, the samples were investigated according to ASTM-D3744 in aggressive Sulfuric Acid attack. Sulfuric Acid solution with a concentration of 5% was used for determining the resistance of samples for a total exposure period of 24 h. The loss in strength was investigated and visual assessment was also evaluated of the degraded samples as shown in Fig. 5.

## Results and discussion

### Compressive strength

**Figure 6:**
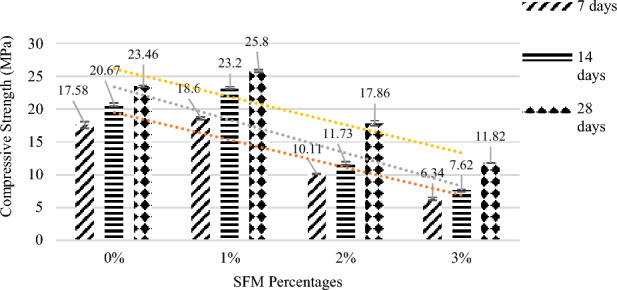
Compressive strength vs SFM percentages.

It is concluded from Fig. 6 and Table 6 that the compressive strength increases as the percentage of SFM increases from 0 to 1% and gives maximum strength by using 1% SFM which can be attributed to the crack-resistant properties or bridging effect of the fibers present in the Facemask^37^. Further increasing the SFM percentage causes a decrease in the strength due to the increasing pores. This variability in the strengths can be shown in percentage as loss or gain in the table below which shows that the strength increases by 9.97% when using the facemask fibers at 1% and 10% silica fume by weight of cement. The increasing facemask percentages to 2% and 3% increases the voids and porosity of concrete making it less dense, hence a decrease in the compressive strength occurred.

**Table 6:** Compressive strength vs SFM percentages.

Mix No.	SFM% (%)	RCA% (%)	Compressive strength (Mpa)		
			7 days	14 days	28 days
1	0	0	17.58	20.67	23.46
2	1	0	18.6	23.2	25.8
3	2	0	10.11	11.73	17.86
4	3	0	6.34	7.62	11.82

The attained compressive strengths of different percentages of SFM showed that the optimum percentage of SFM is 1% that gave us maximum compressive strength, In further trials, 1% SFM and different percentages of RCA along with 10% additional Silica Fume by weight of cement were used.

**Figure 7:**
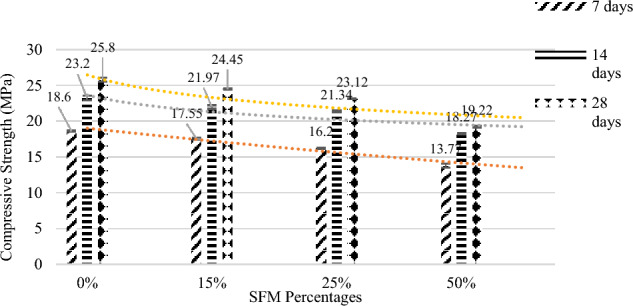
Compressive strengths vs RCA percentages.

It is concluded from Fig. 7 in which the error bars has also been incorporated based on the standard deviation and Table 7 that 1% SFM and 0% RCA have given maximum compressive strength of 25.8 MPa. Increasing RCA percentages from 0 to 50% leads to a decrease in strength which can be attributed to the weak and porous nature of RCA as compared to natural aggregate, but all these strengths lie within the allowable limits for Rigid Pavement i.e. minimum 20 MPa. So, to move to the economical side, a high percentage of RCA ≤ 50% can be used.

**Table 7:** Compressive strength vs RCA percentages.

Mix No.	SFM% (%)	RCA% (%)	Compressive strength (Mpa)		
			7 days	14 days	28 days
1	1	0	18.6	23.2	25.8
2	1	15	17.55	21.97	25.45
3	1	25	16.2	21.34	23.12
4	1	50	13.77	18.27	19.22

### Splitting tensile strength

**Figure 8:**
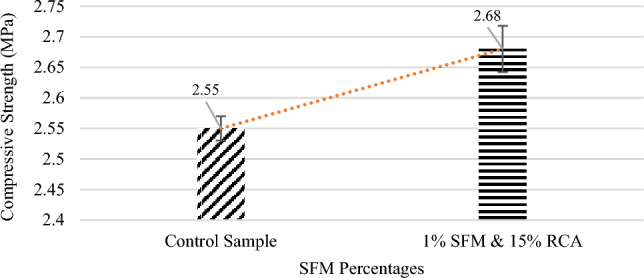
Split tensile strength vs SFM percentage.

The specimen with the highest compressive strength in Table 7 was subjected to loading and compared with the control specimen. From Fig. 8 it can be concluded that split tensile strength increased effectively as compared to that of the normal sample having no SFM fibers, this increase in strength was attributed to the reinforcing, crack resistance, and bridging effect caused by SFM fibers^37,38^. This improvement of tensile strength is also due to more flexibility of SFM fibers as compared to RCA particles as these fibers are classified as short and discontinuous fibers which could provide good results in terms of strength and stiffness.

### Durability test

**Figure 9:**
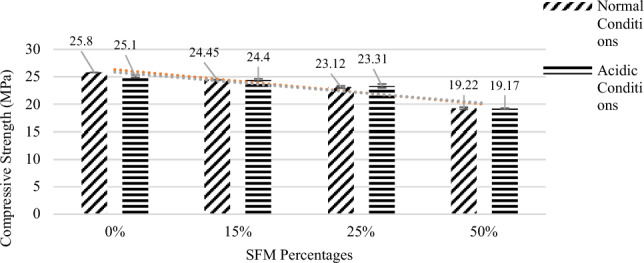
Durability of various specimens containing various RCA percentages.

Concrete samples were examined under a harsh sulfuric acid attack to determine their durability. Test samples having RCA at 0%, 15%, 25%, and 50% were compared to control samples, which had SFM at the optimal amount. For a total exposure time of 24 h, samples were exposed to a 5% sulfuric acid solution to test their resistance. Investigation of the strength loss and evaluation of the degraded samples based on visual inspection was also conducted. The results of the durability test were reported in Fig. 9 and Table 8. It was found that no particular strength loss occurred. The maximum strength loss was 0.7 MPa out of 25.8 MPa which is not a major issue. During the visual inspection, the concrete color slightly changed from normal color to light color as shown in Figure as the research carried out by Anish Banerjee also indicates that concrete color deteriorates in acidic environment^39^.

**Table 8:** Durability test results.

Trial No.	SFM% (%)	RCA% (%)	Compressive strength of 28 days	
			Normal condition	Acidic condition
1	1	0	25.8	25.10
2	1	15	24.45	24.40
3	1	25	23.31	23.12
4	1	50	19.22	19.17

### Comparison with previous studies

In comparison to regular concrete, fiber concrete has advantages in terms of improving its compressive strength, split tensile strength, and ductility. According to previous research carried out by *Cory High*, the use of combined chopped basalt fiber and basalt fiber reinforced bars as additives caused a maximum increase in compressive strength of concrete at the age of 28 days up to 6% ^40^. Another research on the basalt fiber was carried out by *Biradar* in which the maximum increase in the compressive strength at the age of 28 days was recorded up to 9% by using 0.3% fibers by volume of concrete^41^. Moreover, research by *Sivakumar* concluded that after using fibers of 0.6 kg/m^3^, the compressive strength for 28 days of Glass fiber reinforced concrete (GFRC), polyester, and nylon improved by 3%, 1.8%, and 6.3% respectively^28^.

The research carried out by *Pelisser* determined that by using polyethylene terephthalate (PET) fibers by volume of concrete the maximum increase in compressive strength is recorded up to 1% at the age of 28 days^43^.

**Figure 10:**
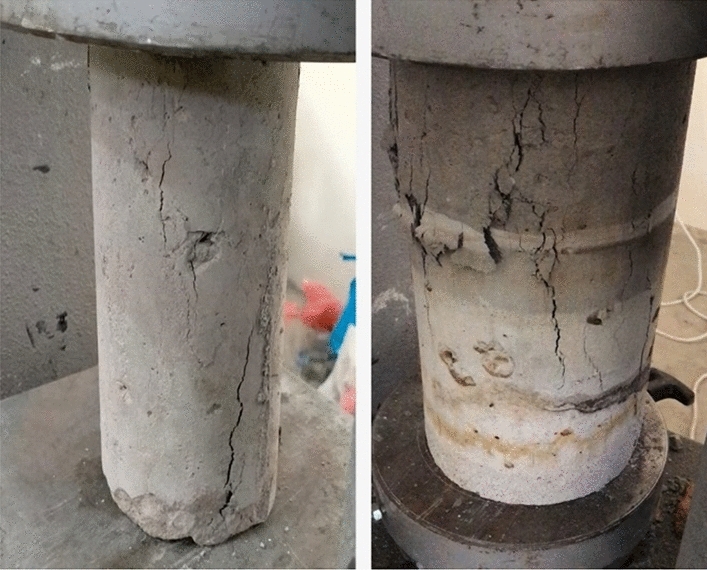
Change of color of the specimens.

**Figure 11:**
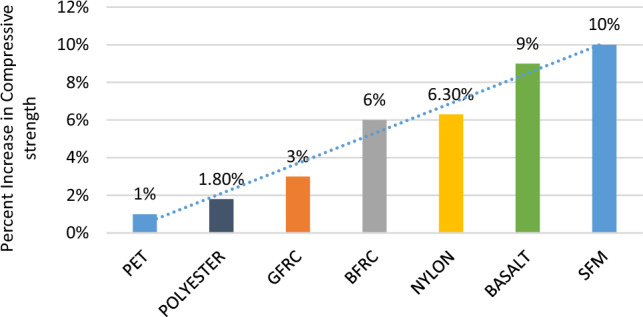
Increase in compressive strength of various fibers percentage.

All of the improvements in strength by using the above-mentioned fibers are less than the increase caused in compressive strength percentage by using 1% SFM fiber by volume of concrete. Due to this enhancement in mechanical properties, ease in availability, environment friendly (trash usage) Fig. 10, and cost-effectiveness of SFM fibers makes it is more suitable to be used in concrete and Rigid Pavement. The results of the previous works have been reported in Fig. 11.

In comparison to control sample, the split tensile strength increased as shown in Fig. 8 and this increase in strength was attributed to the reinforcing, crack resistance, and bridging effect caused by SFM fibers^44^. This increase in split tensile strength due to SFM fibers has also been observed by Shannon Kilmartin-Lynch^8^.

In comparison of the control sample of durability test, it was found out that specimen lost 0.7 MPa strength out of 25.8 Mpa. The total strength loss was recorded 2.71% when the specimens were exposed to 5% sulphuric acid solution. The research of Arash Arjomandi also indicates that with the rise in the time of exposure to acidic environment, the concrete strength declines as he also used 5% sulphuric acid solution to find durability of concrete having steel fibers and nylon granules^45^.

## Conclusion

- From this study, it can be concluded that to control COVID-19 waste and due to non-biodegradable nature of the material of the SFM, it has been added to concrete in different percentages.
- Among all the SFM percentages (0–3%), 1% SFM gives maximum compressive strength. However, a further increase in SFM percentage decrease the compressive strength.
- To decrease construction demolition waste, Recycled Concrete Aggregates (RCA) is introduced to the mix in different percentages and Silica fume is used 10% by weight of cement in addition to counter the decrease in strength caused by RCA in concrete.
- By introducing RCA (15%, 25%, and 50%) to the mix, the compressive strength decreased with the increasing percentages of RCA, but all these strengths lie within the limits of the required strength of rigid pavements i.e. 20Mpa.
- Among all these samples having different percentages of SFM and RCA, samples having 1% SFM and 15% RCA gave the highest compressive strength.
- SFM fibers increased the split tensile strength as compared to the sample having no SFM fibers, further increase of SFM fibers in the mix can increase split tensile strength but can cause a decrease in compressive strength.
- Samples having 1% SFM and 15%, 25%, and 50% RCA were found durable as no significant decrease in strengths was observed when compared to the control sample.
- It is estimated that for a 1 km rigid pavement having 48 feet in width and 6 inches in thickness if we use 1% SFM and 15% RCA, it will consume 9.8 tons of SFM fibers and 382.5 tons of RCA.

## Recommendations

The recommendations for further research on the same topic are as follows.

1. Consideration for effects of different sizes of facemasks (aspect ratio) either short fibers or long fibers and their outcome on mechanical properties of concrete should be considered.
2. Evaluation of the effects of the use of SFM on the water absorption, moisture susceptibility modulus, and distribution of SFM fibers in the mixture for quality control.
3. Evaluation of the density variability due to the use of these facemask fibers and RCA to predict durability, compressive strength, porosity, and water absorption of the concrete.
4. Evaluation of SFM percentages in between 0 and 1% and studying its effects on mechanical properties of concrete.

## Data Availability

The data will made available on a reasonable request to the corresponding author.
